# Microscopic Dynamics and Topology of Polymer Rings Immersed in a Host Matrix of Longer Linear Polymers: Results from a Detailed Molecular Dynamics Simulation Study and Comparison with Experimental Data

**DOI:** 10.3390/polym8080283

**Published:** 2016-08-04

**Authors:** George D. Papadopoulos, Dimitrios G. Tsalikis, Vlasis G. Mavrantzas

**Affiliations:** 1 Department of Chemical Engineering, University of Patras and FORTH-ICE/HT, Patras, GR 26504, Greece; georgepapa5@chemeng.upatras.gr (G.D.P.); tsalikis@chemeng.upatras.gr (D.G.T.); 2 Department of Mechanical and Process Engineering, Particle Technology Laboratory, ETH-Z, CH-8092 Zürich, Switzerland

**Keywords:** blend, molecular dynamics, poly(ethylene oxide), ring, simulation, threadings, topological analysis, probe

## Abstract

We have performed molecular dynamics (MD) simulations of melt systems consisting of a small number of long ring poly(ethylene oxide) (PEO) probes immersed in a host matrix of linear PEO chains and have studied their microscopic dynamics and topology as a function of the molecular length of the host linear chains. Consistent with a recent neutron spin echo spectroscopy study (Goossen et al., *Phys. Rev. Lett.* 2015, *115*, 148302), we have observed that the segmental dynamics of the probe ring molecules is controlled by the length of the host linear chains. In matrices of short, unentangled linear chains, the ring probes exhibit a Rouse-like dynamics, and the spectra of their dynamic structure factor resemble those in their own melt. In striking contrast, in matrices of long, entangled linear chains, their dynamics is drastically altered. The corresponding dynamic structure factor spectra exhibit a steep initial decay up to times on the order of the entanglement time *τ*_e_ of linear PEO at the same temperature but then they become practically time-independent approaching plateau values. The plateau values are different for different wavevectors; they also depend on the length of the host linear chains. Our results are supported by a geometric analysis of topological interactions, which reveals significant threading of all ring molecules by the linear chains. In most cases, each ring is simultaneously threaded by several linear chains. As a result, its dynamics at times longer than a few *τ*_e_ should be completely dictated by the release of the topological restrictions imposed by these threadings (interpenetrations). Our topological analysis did not indicate any effect of the few ring probes on the statistical properties of the network of primitive paths of the host linear chains.

## 1. Introduction

Present-day research in the dynamics of ring polymers [1,2,3,4,5,6,7,8,9,10,11,12,13,14] varies from experimentally sophisticated measurements of properties such as dynamic structure factor, viscosity, and stress relaxation [15,16,17,18,19,20,21,22,23] to new theoretical developments [24] beyond the animal picture and to computer simulations [25,26,27,28,29,30,31,32,33,34,35,36,37] based upon either detailed, atomistic models or simpler but useful coarse-grained representations. An example of the first type of research is the investigation of the rheological properties of highly purified ring polymer melts in the crossover region from unentangled to entangled [22,23] and of their microscopic dynamics using neutron spin echo spectroscopy (NSE) and pulse field gradient NMR [17,19,20,21]. Small angle neutron scattering (SANS) experiments [21] are also used to study structural properties as well as conformational deviations from the unperturbed Gaussian model. An example of the second type of research is the development of new scaling models of self-similar conformations [24]. An example of the third type of research is the performance of computer simulations with model, non-concatenated ring polymers (in solution, melt and blends with linear chains) using well-validated atomistic force-fields to identify ring–ring and ring–linear interpenetrations [35,37].

Overall, ring polymers exhibit material properties that are very different from those of the other two classes of polymers, linear and branched, of the same molecular length due to the absence of chain ends and their closed-loop structure. Atomistic molecular dynamics (MD) simulations, e.g., with cyclic and linear polyethylene (PE) melts, have shown that, for short chain lengths, ring PE melts exhibit a slower center-of-mass diffusivity than their linear analogs; but as the chain length increases, a crossover is observed above which the chain center-of-mass diffusivity of the rings becomes faster than of the linear melts [27,29]. Short chain-length cyclic alkanes, in particular, exhibit properties typical of semiflexible polymers due to the fact that their conformation space is dominated by specific conformers [7]. Von Meerwall et al. [12] have argued that these semiflexible (or even rigid) structures can explain why the diffusion coefficient of cyclic alkanes as a function of their molecular weight adheres to a power-law behavior (thus deviating significantly from the Rouse scaling).

Of relevance to our contribution here is the recent NSE study of Goossen et al. [21] which revealed that the segmental dynamics of polymer rings immersed in linear chains is fully controlled by the host, which in turn renders rings as ideal probes for sensing the dynamics of the long linear matrix chains through their effect on the long-time dynamics of the rings; this reveals immediately the entanglement spacing. Previous computer simulation studies [32,33,34,35,36,37] have addressed the opposite effect, namely the influence of the presence of a small number of linear chain contaminants on the long-time dynamics and viscoelastic properties of melts of long polymer rings.

Both experimental measurements and computer simulations provide convincing evidence that the long-time behavior of polymer rings is controlled by strong topological interactions related either with ring–ring or with linear–ring threading. It appears that linear–ring interpenetrations are, on average, active for significantly longer times than ring–ring ones, thus they have a stronger impact on ring dynamics [37]. One can envisage that ring–ring and ring–linear disentanglements are akin to a chain escaping a network, hence to a first approximation their effect on ring melt dynamics can be described by the same mathematical expressions of reptation theory as for melts of entangled linear chains [38,39,40,41] albeit with different effective plateau moduli and disentanglement times. It turns out, in particular, that a modified expression for the stress relaxation modulus *G*(*t*) of polymer rings accounting for these extra contributions provides an accurate description of experimental rheological data for several ring polymer melts [37].

Motivated by the recent NSE measurements of Goossen et al. [21], in this work we have performed atomistic MD simulations of model polymer blends consisting of a small number of ring poly(ethylene oxide) (PEO) molecules in a PEO matrix at a very small concentration in the ring component (weight fraction of rings ϕ = 0.1). Our scope is to investigate the effect of the molecular length of the host linear chains on the conformation and dynamics of the probe rings and, through this, to conclude about the capability of rings to sense the dynamics and topology of their surrounding linear chains (unentangled or entangled). Consistent with the measurements of Goossen et al. [21], we will see that the dynamics of rings in infinite dilution in the matrix of linear chains is drastically altered when the linear chains are entangled causing the spectra of their dynamic structure factor to approach quickly constant asymptotic values, following a very steep initial time decay on scales that are commensurate with the entanglement time *τ_e_* of linear chains at the same temperature.

The structure of our paper is as follows. In Section 2, we describe the systems (melts and blends) that were simulated in this work and provide some technical details about the simulation methodology and the atomistic force-field used to perform the simulations. In Section 3, we present the simulation results and how they compare with the corresponding experimental measurements [21] or other findings from previous simulation studies. Our paper ends with Section 4 summarizing our conclusions and discussing possible future plans.

## 2. Molecular Model and Simulated Systems

The ring PEO chains considered in our simulations are represented by the formula –CH_2_–O–(CH_2_–CH_2_–O)*_N_*–CH_2_– while the linear PEO chains by the formula CH_3_–O–(CH_2_–CH_2_–O)*_N_*–CH_3_. Guided by the experimental work of Goossen et al. [21], the ring molecules were taken to consist of *N* = 456 monomers per chain corresponding to a molecular weight of 20,064 g/mole (so, for brevity, we will denote them as PEO-20k rings), while for the linear chains we considered three different chain sizes, *N* = 41, 228 and 456, corresponding to molecular weights equal to 1806 g/mole (the L-02k matrix), 10,034 g/mole (the L-10k matrix) and 20,066 g/mole (the L-20k matrix). The weight fraction of rings in all three different blends was kept small, equal to ϕ = 0.1, see Table 1. Also shown in Table 1 are the numbers of ring and linear chain molecules considered for each system in the simulation cell.

For comparison, we have also simulated the following pure melts: (a) a pure ring PEO-20k melt consisting of 38 ring molecules; (b) a pure linear PEO-02k melt consisting of 125 chains; (c) a pure linear PEO-10k melt consisting of 44 chains; and (d) a pure linear PEO-20k melt consisting of 38 chains in the simulation cell.

In all MD simulations, we used the modified TrAPPE united-atom (UA) force-field introduced by Fischer et al. [42,43], which has been thoroughly validated through extensive comparisons of its predictions for several material properties (density, mean square radius-of-gyration, self-diffusion coefficient, dynamic structure factor, etc.) of low and high molecular weight linear and ring PEO melts with published simulation [44] or experimental [45,46,47,48,49,50,51,52] data and/or the predictions of other force fields reported in the literature [53]. The simulations were executed with the GROMACS [54] simulation software in the isothermal–isobaric (NPT) statistical ensemble by applying the Nosé–Hoover thermostat [55,56] coupled with the Parrinello–Rahman [57] barostat to keep the temperature *T* and the pressure *P* fixed at the desired values (*T* = 413 K and *P* = 1 atm). As it can be inferred from Table 1, large cubic simulation cells (subject to periodic boundary conditions) were employed in all simulations (containing in some cases over 720 chains and more than 10^5^ atoms) to minimize finite system size effects. The equations of motion were integrated using a single time step equal to 2 fs.

Initial configurations were created using the commercially available software platform MAPS [58] following a systematic approach that avoids ring–ring concatenation and self-knotting. All technical details regarding system preparation can be found elsewhere [33]. The atomistic configurations were subjected to potential energy minimization in order to remove undesired atom–atom overlaps, and then very long MD simulation runs, longer than about 2 μs, were carried out to ensure complete equilibration of the structural and conformational properties at all length scales, and thus the attainment of reliable predictions for all material properties of interest, especially those referring to long time dynamics.

To investigate the topological properties of the simulated blends, the large trajectories accumulated in the course of the long MD runs were subjected to a primitive path (PP) analysis using the CReTA (Contour Reduction Topological Analysis) algorithm [59] properly extended [35] to account for the absence of chain ends in the ring molecules. CReTA allows reducing the classical atomistic trajectory to a trajectory of PPs. The PP is defined as the shortest multiple piecewise continuous path that connects the two end points of the chain that are considered to be fixed in space, and which has the same topology as the original atomistic chain relative to topological constraints imposed by the neighboring chains. In the course of CReTA, the contour length of a chain (which is represented as a series of fused spheres) is continuously shrunk through the application of random aligning string moves by simultaneously accounting for hard core interactions to prevent chain crossing and preserve system topology. The algorithm results in an ensemble of shortest paths for all chains in the simulation cell in the form of connected strands that come close to each other at certain points, typically referred to as entanglement points or kinks.

At the end of the topological analysis with CReTA, the ensemble of atomistic chains has been mapped to an ensemble of PPs. Linear chains have been reduced to sequences of straight entanglement strands and the same for ring molecules which are observed to have the shape of 3d polygons due to their closed-loop structure. In our analysis of topological interactions, the reduction to ensembles of PPs by CReTA was followed by a detailed geometric analysis based on ring triangulation and vector calculus in order to identify all ring–linear [35] and ring–ring [37] interpenetrations (the so-called threading events) in the blends and in the pure ring melts. Results from all of the above studies are presented in the next Section.

## 3. Results and Discussion

### 3.1. Conformational Properties

**Figure 1:**
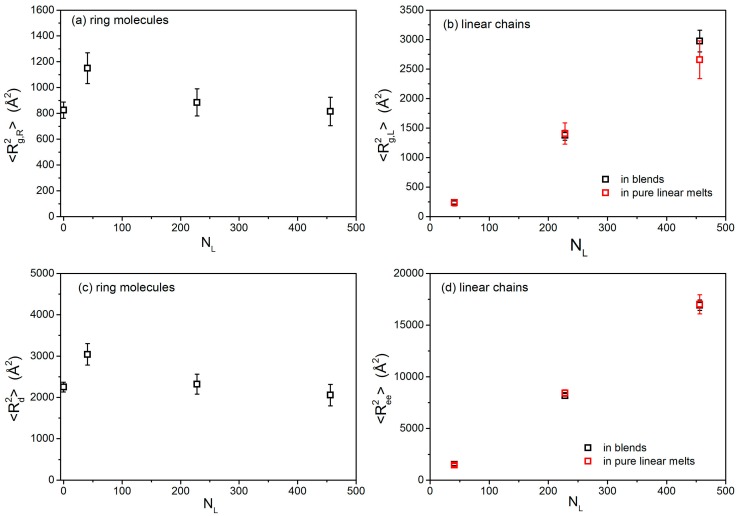
Dependence of: (**a**) $\langle R_{g,R}^{2}\rangle$, (**b**) $\langle R_{g,L}^{2}\rangle$, (**c**) $\langle R_{d}^{2}\rangle$ and (**d**) $\langle R_{\mathrm{ee}}^{2}\rangle$ on the chain length *N*_L_ (L stands for linear) of the host linear chains. Results are also shown for the same quantities in the corresponding pure melts.

Conformational properties of ring and linear PEO molecules in the three simulated blends have been analyzed in terms of their mean-square chain radius of gyration $\langle R_{g,R}^{2}\rangle$ (R stands for ring) and $\langle R_{g,L}^{2}\rangle$ (L stands for linear), respectively. For ring molecules, we have also calculated their mean-square diameter distance $\langle R_{d}^{2}\rangle$, while for linear chains we have calculated the mean-square chain end-to-end distance $\langle R_{\mathrm{ee}}^{2}\rangle$. How the four different measures vary from blend to blend is shown in Figure 1 (the numerical data are also reported in Table 2), where for comparison we also show the values of $\langle R_{g,R}^{2}\rangle$, $\langle R_{g,L}^{2}\rangle$, $\langle R_{d}^{2}\rangle$ and $\langle R_{\mathrm{ee}}^{2}\rangle$ in the corresponding pure (ring or linear) melts. Error bars have been calculated by using the technique of block averaging for the equilibrated part of the simulation trajectory. For pure ring or linear PEO melts, simulation results [25,26,27,30,35] and experimetal data [13,20] agree in that the respective average radii-of-gyration scale with the number *N* of monomers per chain as $\langle R_{g,R}^{2}\rangle \sim N^{0.83}$ and $\langle R_{g,L}^{2}\rangle \sim N^{1}$, respectively. The different scalings are a direct manifestation of the more compact structure of pure ring polymers compared to the equivalent linear polymers which for all practical purposes behave as Gaussian coils in their own melt.

Figure 1 shows that the value of $\langle R_{g,R}^{2}\rangle$ of the few PEO-20k rings present in the three blends with linear chains depends strongly on the size of the linear chains. We also notice that the dependence is not monotonic. $\langle R_{g,R}^{2}\rangle$ is highest (by approximately 36% compared to its value in the pure ring PEO-20k melt) in the L-02k blend and then decreases. That is, in the L-02k blend, ring chains are significantly expanded. On the other hand, in the two higher chain length (entangled) blends, L-10k and L-20k, the size of rings deviates only by very little compared to the value in their own ring melt. Clearly, the variation of the ring size with the molecular length of the host linear chain matrices is unexpected but can be understood in terms of the degree of their threading by linear chains (see Section 3.6), which causes ring chains to swell. In particular, we keep in mind that we are comparing the various blends at the same mole or weight fraction of rings, which implies that the number of linear chains present in the blend decreases proportionally with their chain length (see Table 1). That is, in the L-02 blend, the population of linear chains is five times larger than in the L-10k blend; and, similarly, in the L-10k blend, the population of linear chains is two times larger than in the L-10k blend. It appears then (see Section 3.6) that a larger number of short linear chains provide more threading than a smaller number of longer ones (at the same weight fraction). This can explain why rings are swollen in the L-02k blend much more than in the L-10k blend, and similarly why rings in the L-10k blend are swollen a little more than in the L-20k blend. More quantitative results for the degree of threading of the ring probes by the host linear chains are provided in Section 3.6.

It is of interest to discuss how these results compare with the experimental conclusions of Goossen et al. [21] through SANS measurements. It turns out, however, that these authors report SANS data for 20k ring PEO molecules in a deuterated linear 20k PEO matrix only at ϕ = 0.01, i.e., at a much smaller concentration than the concentration (ϕ = 0.1) at which the MD simulations were performed here. At this volume fraction (ϕ = 0.01), Goossen et al. [21] report that rings in the linear matrix assume unperturbed Gaussian conformations that are significantly expanded compared to the rather compact structure in their own melt. Their estimate was that the radius of gyration of the 20k PEO rings was 36.5 Å, which is 20% larger than the corresponding value of 30.5 Å in the pure ring melt. Our MD simulations do not indicate any change in the size of rings in the L-20k blend at ϕ = 0.10.

In contrast to rings, the conformational properties of the linear matrix chains appear to remain practically unaffected by the presence of the few ring molecules (any differences in Table 2 are within the error bars of the simulation data). We also see that, for the pure linear melts, $\langle R_{g,L}^{2}\rangle$ varies linearly with *N*, in agreement with the Gaussian hypothesis for long linear polymers. Analysis of the $\langle R_{d}^{2}\rangle$ and $\langle R_{\mathrm{ee}}^{2}\rangle$ data in Figure 1c,d yields similar conclusions.

We also observe that the ratio $\langle R_{\mathrm{ee}}^{2}\rangle /\langle R_{g,L}^{2}\rangle$ for the linear chains in all melts studied in this work is around 6, which is another indication that, to an excellent approximation, linear PEO chains obey the random-coil hypothesis.

We have also calculated the persistence length $l_{p}$ and the packing length $\tilde{l}$ of the simulated ring and linear PEO systems (in their own melt and in the three blends). The persistence length $l_{p}$ was computed by using equation $\langle u\left(s\right)\cdot u\left(0\right)\rangle =\exp\left[-\frac{s}{l_{p}}\right]$, i.e., by calculating the autocorrelation function of the tangent unit vector **u**(*s*) as a function of contour length *s* along the chain from one of its two end points and fitting the resulting curve with an exponential function. The packing length $\tilde{l}$, on the other hand, was computed through $\tilde{l}=\frac{M}{\rho N_{\mathrm{Av}}\langle R_{g}^{2}\rangle }$ where *M* is the molecular weight, *ρ* is the density, *N*_Av_ the Avogadro number and $\langle R_{g}^{2}\rangle$ the mean-square radius-of-gyration. The simulation results for $l_{p}$ and $\tilde{l}$ are shown in Table 3. The packing length of ring molecules in their own melt is significantly larger (by almost three times) than that of linear chains, a direct manifestation of the more compact structure of rings. We also note that the packing length of the guest PEO 20k ring molecules attains its smallest value in the L-02k blend and its largest value in the L-20k blend. In addition, according to Table 3: (a) the persistence length of linear PEO chains is independent of their molecular length (at least for the chain lengths studied here); (b) the persistence length of linear PEO chains is slightly larger than that of ring molecules; and (c) the persistence length of both ring molecules and linear chains is the same (within the statistical uncertainty of the simulation results) in all systems studied here, either pure melts or blends.

**Figure 2:**
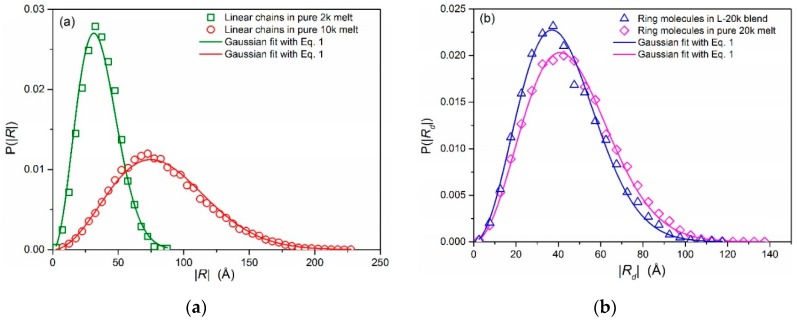
MD simulation predictions (symbols) for the probability distribution function of: (**a**) the magnitude of the end-to-end distance vector for the pure linear PEO-02k and PEO-10k melts; and (**b**) the magnitude of the diameter vector for the ring PEO-20k molecules in their own melt and in the L-20k blend. For comparison (lines), we also show the distributions according to the analytical expression, Equation (1), using the values of $\langle R^{2}\rangle$ and $\langle R_{d}^{2}\rangle$ computed from the MD simulations.

The conformation of linear PEO chains in all simulated systems is accurately described by the Gaussian model. This can be verified in Figure 2a showing the probability distribution function for the magnitude of the chain end-to-end distance vector of the PEO-02k and PEO-10k linear chains examined here (in their own pure melt and in their blends with the few ring probes) as calculated directly from the simulations (symbols) and as predicted by the Gaussian model (lines) according to which:

(1) $$p(R)=\frac{4\pi R^{2}}{{\left(\frac{2}{3}\pi \langle R^{2}\rangle \right)}^{\frac{3}{2}}}\exp\left[-\frac{3R^{2}}{2\langle R^{2}\rangle }\right]$$

Excellent agreement is observed in all cases. It is surprising though that the Gaussian model can also describe the conformation of the ring molecules. This can be verified in Figure 2b showing the probability distribution function of the magnitude of the ring diameter vector for the 20k PEO ring molecules in their own pure melt and in the L-20k blend, as calculated directly from the MD simulations and as predicted by the corresponding Gaussian model, Equation (1), above, suitably adapted for rings (i.e., by replacing the end-to-end vector **R** of linear chains by the diameter vector **R**_d_ of rings). Recently, Ge et al. [24] have proposed a scaling model of self-similar conformations for the size of nonconcatenated entangled ring polymers. Accounting for topological constraints through such a model forces these ring polymers into compact conformations with fractal dimension *d*_f_ = 3 (also called fractal loopy globules).

### 3.2. Normal Mode Analysis

A detailed review of the Rouse model for ring polymers has appeared in [14,27]. Due to the absence of chain ends, odd modes disappear and only even modes contribute to the dynamics:

(2) $$R_{n}(t)=X_{0}+\sum_{p:\mathrm{even}}^{\infty }\left[2X_{p}\cos\left(\frac{p\pi n}{N}\right)+2Y_{p}\sin\left(\frac{p\pi n}{N}\right)\right]$$

(3) $$X_{p}(t)=\frac{1}{N}\int_{0}^{N}R_{n}(t)\cos\left(\frac{p\pi n}{N}\right)dn\;\mathrm{for}\;p=0,\;2,\;4,\;6,\cdots$$

(4) $$Y_{p}(t)=\frac{1}{N}\int_{0}^{N}R_{n}(t)\sin\left(\frac{p\pi n}{N}\right)dn\;\mathrm{for}\;p=\;2,\;4,\;6,\;8,\cdots$$

In the above expressions, $R_{n}(t)$ indicates the position of the *n*-th bead along the ring, *p*:even accounts for the even modes (i.e., p=2,  4,  6,⋯), $X_{p}(t)$ is the *p*-th cosine mode for p= 2,  4,  6,  8,⋯, and $Y_{p}(t)$ is the *p*-th sine mode for p= 2,  4,  6,  8,⋯. According to the Rouse model, the normal coordinates satisfy the following autocorrelation functions:

(5) $$\langle X_{p\alpha }(t)X_{q\beta }(0)\rangle =\delta_{\mathrm{pq}}\delta_{\alpha \beta }\frac{k_{B}T}{k_{p}}\exp\left(-t/\tau_{p}\right)\;\mathrm{for}\;p,q=0,\;2,\;4,\;6,\cdots$$

(6) $$\langle Y_{p\alpha }(t)Y_{q\beta }(0)\rangle =\delta_{\mathrm{pq}}\delta_{\alpha \beta }\frac{k_{B}T}{k_{p}}\exp\left(-t/\tau_{p}\right)\;\mathrm{for}\;p,q=2,\;4,\;6,\cdots$$

(7) $$\langle X_{p\alpha }(t)Y_{q\beta }(0)\rangle =0\;\mathrm{for}\;\mathrm{all}\;p\;\mathrm{and}\;q$$

where

(8) $$k_{p}=\frac{6\pi^{2}k_{B}T}{Nb^{2}}p^{2}\;\mathrm{for}\;p=0,\;2,\;4,\;6,\cdots$$

denotes the magnitude (strength) of the *p*-th normal mode, and

(9) $$\tau_{p}=\frac{2N\zeta }{k_{p}}=\frac{\zeta N^{2}b^{2}}{3\pi^{2}k_{B}T}\frac{1}{p^{2}}=\frac{4\tau_{2}}{p^{2}}\;\mathrm{for}\;p=\;2,\;4,\;6,\;8,\cdots$$

the corresponding relaxation time. In the above equations, *ζ* is an effective hydrodynamic friction coefficient, *k* the modulus (constant) of the Gaussian spring connecting successive beads along the ring, *k*_B_ the Boltzmann constant, *T* the temperature, and *b*^2^ the average mean-square-distance between adjacent beads at equilibrium. The longest relaxation (Rouse) time for a ring molecule is therefore

(10) $$\tau_{R,\mathrm{ring}}=\tau_{2}=\frac{\zeta N^{2}b^{2}}{12\pi^{2}k_{B}T}$$

which is equal to one fourth of the corresponding Rouse time for a linear chain comprising the same number of beads *N* ($\tau_{R,\mathrm{linear}}=\tau_{1}=\zeta N^{2}b^{2}/3\pi^{2}k_{B}T$); i.e., $\tau_{R,\mathrm{ring}}=\tau_{R,\mathrm{linear}}/4$.

**Figure 3:**
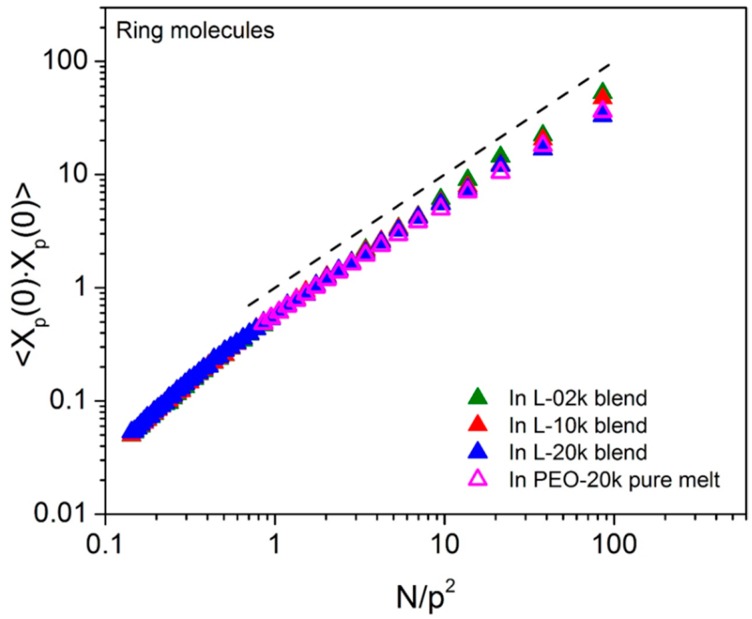
The squared amplitudes of the Rouse normal modes $\langle X_{p}{\left(0\right)}^{2}\rangle$ for the ring molecules in the three blends and in a pure ring PEO-20k melt as a function of Np2 in a log–log plot. The dashed line has been drawn with a slope of 1 as a guide for the eye and corresponds to the Rouse scaling.

The information about the microscopic dynamics accumulated in the course of the long MD simulations of the systems carried out in this work allows us to test the degree to which ring dynamics deviates from the Rouse behavior in their blends with linear chains of different chain length. In Figure 3, we show results for the mean-squared amplitude $\langle X_{p}{\left(0\right)}^{2}\rangle$ of the *p*-th Rouse mode with mode number *p*. As suggested by Equations (5) and (8), if ring dynamics were Rouse-like, $\langle X_{p}{\left(0\right)}^{2}\rangle$ would vary with *p* as $\frac{1}{p^{2}}$. Our simulations (see Figure 3) suggest that, for the PEO-20k ring molecules studied here, the Rouse scaling in their own melt but also in the three blends is followed only for the lower *p* modes (more precisely for the modes *p* for which $\frac{N}{p^{2}}>1$); for higher *p* modes (i.e., for $\frac{N}{p^{2}}<1$), systematic deviations are observed from the Rouse theory due (most likely) to larger deviations of ring conformations at short length scales (compared to longer length scales) from the Gaussian model.

**Figure 4:**
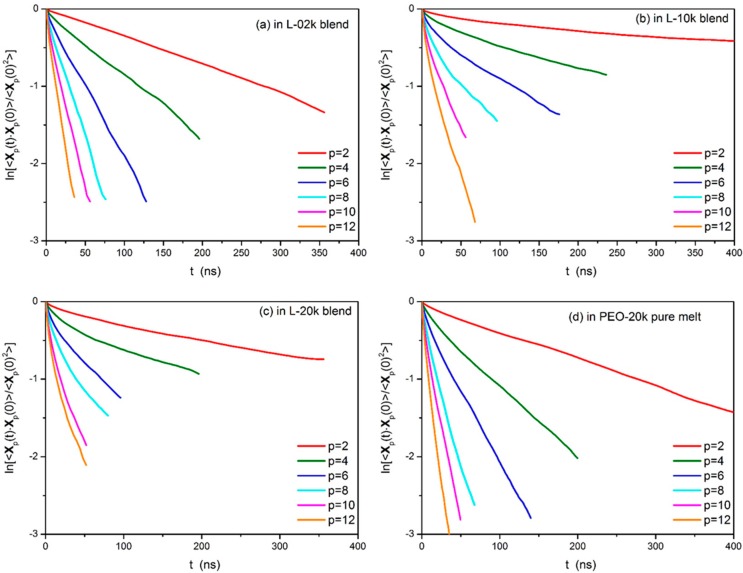
Log-linear plots of the normalized time autocorrelation functions of several Rouse modes for the PEO-20k ring molecules in: (**a**) the L-02k blend, (**b**) the L-10k blend, (**c**) the L-20k blend, and (**d**) the pure ring PEO-20k melt.

We have further examined how the time decay of the normalized autocorrelation function $\frac{\langle X_{p}(t)\cdot X_{p}(0)\rangle }{\langle X_{p}{\left(0\right)}^{2}\rangle }$ for p= 2,  4,  6,  8,⋯ of the probe PEO-20k ring chains in the three blends changes compared to the corresponding time decay in their own melt. According to Equation (5), a log-linear plot of $\frac{\langle X_{p}(t)\cdot X_{p}(0)\rangle }{\langle X_{p}{\left(0\right)}^{2}\rangle }$ versus *t* for each of the simulated melts should yield a straight line. Numerical results for the relaxation of the first six modes (*p* = 2, 4, 6, 8, 10 and 12) from our simulations with the four systems (the three blends and the pure ring melt) are shown in Figure 4. We see that, to a good degree, except for very short times, the decay of normal modes in the pure ring melt and in the L-02k blend at long enough times is exponential-like, i.e., it exhibits Rouse-like characteristics. In contrast, in the L-10k and L-20k blends (especially in the latter), the Rouse features are lost. Ring dynamics in matrices of entangled linear chains is definitely not Rouse-like.

**Figure 5:**
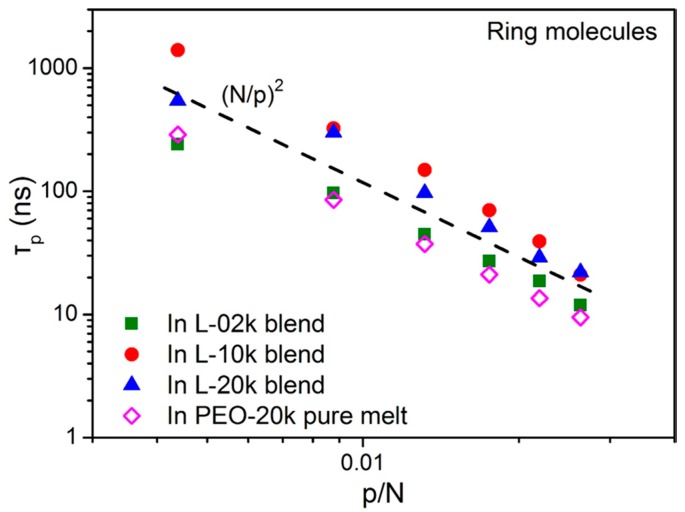
The scaling of the characteristic times $\tau_{p}$ describing the relaxation of the Rouse normal modes *p* with chain length *N*. The dashed line indicates the Rouse model, namely, τp~(Np)2, and has been drawn as a guide for the eye.

In Figure 5, we show the scaling of the characteristic times $\tau_{p}$ describing the relaxation of the *p*-th mode with mode number *p* in the four systems (the three blends and the pure ring melt) after computing the integrals below each curve of Figure 4 numerically. The dashed line in the figure indicates the Rouse scaling, Equation (9). Our MD data indicate that ring molecules in their own melt follow the Rouse scaling quite closely in the sense that the slope of the curve is −2 (with the exception of the smallest modes where the data show some bending towards the low *p* values), namely,

(11) τp∝(Np)2.0

which is identical to the Rouse scaling, see Equation (9). This is not the case, however, in the three blends. With the exception of the unentangled blend (L-02k) where the characteristic relaxation times for all modes *p* are close to the corresponding relaxation times in the pure ring melt (either slightly above at the higher *p* modes or slightly below at the lower *p* modes), in the other two blends (L-10k and L-20k), all characteristic relaxation times *τ*_p_ are well above those corresponding to the pure PEO-20k ring melt. This demonstrates that dynamics of rings in these entangled blends is significantly slower than in their own melt at all length scales. Indeed, for the L-10k and L-20k blends, the slope is more or less around 2, but all points are shifted upwards.

Our MD results suggest that we can distinguish between two types of dynamics: unrestricted Rouse motion at all length scales in the pure ring and in the L-02k blend, and restricted dynamics in the L-10k and L-20k blends which most likely should be attributed to strong ring–ring and ring–linear threading events [35,37]. We will discuss this issue further in Section 3.6 where we will present the results of our geometric (topological) analysis for ring threading by linear chains and its dependence on linear chain length. According to Figure 5, the effect of restricted dynamics in the two entangled blends (L-10k and L-20k) is more pronounced at smaller *p* values, i.e., at longer length scales. This suggests that a possible way to correct for the deviations (and thus to improve the comparison between simulation predictions and Rouse model for ring dynamics in the two entangled blends) could be by using an increased friction coefficient for the smaller modes. We hope to be able to present such a model in the future.

### 3.3. Dynamic Structure Factor

**Figure 6:**
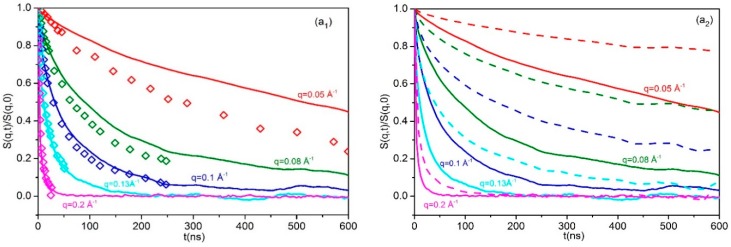

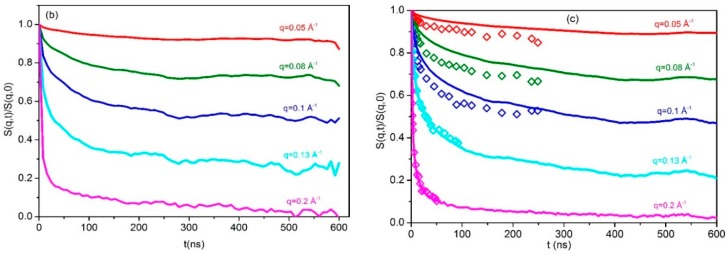
Computed (continuous or dashed lines) and experimentally measured (diamonds) $\frac{S(q,t)}{S(q,0)}$ plots for several wavenumbers *q*, for: (**a_1_**) ring molecules in the L-02k blend together with data from Reference [21]; (**a_2_**) ring molecules in the L-02k blend and in the pure PEO-20k ring melt (dashed lines); (**b**) ring molecules in the L-10k blend; and (**c**) ring molecules in the L-20k blend together with data from Reference [21].

In our MD simulations, the dynamic structure factor $\frac{S(q,t)}{S(q,0)}$ was computed through

(12) $$\frac{S(q,t)}{S(q,0)}=\frac{\sum_{n=1}^{N}\sum_{m=1}^{N}\langle \sin[qR_{\mathrm{nm}}(t)]/qR_{\mathrm{nm}}(t)\rangle }{\sum_{n=1}^{N}\sum_{m=1}^{N}\langle \sin[qR_{\mathrm{nm}}(0)]/qR_{\mathrm{nm}}(0)\rangle }$$

and the results for all blends are shown in Figure 6. In Equation (12), *q* is the magnitude of the scattering vector **q**, $R_{\mathrm{nm}}\left(t\right)$ denotes the magnitude of the displacement vector $R_{\mathrm{nm}}\left(t\right)=R_{n}\left(t\right)-R_{m}\left(0\right)$ between chain segments *n* and *m*, and *N* is the number of atomistic units along the chain. In Figure 6a–c, the MD predictions for $\frac{S(q,t)}{S(q,0)}$, for several scattering vector magnitudes *q* (= 0.05, 0.08, 0.1, 0.13, and 0.2 Å^−1^) are the solid and dashed lines, while the open symbols are the experimentally measured NSE spectra [21] for the L-02k and L-20k systems. According to Figure 6:

(a) In the short L-02k blend (Figure 6a1), the computed $\frac{S(q,t)}{S(q,0)}$ curves for all *q*’s decay monotonically and smoothly over short and long time scales (that extend up to 600ns). The rate of decay is steeper at short times and deceases as the time increases, but in a very smooth way. As a result, only quantitative differences are observed from the corresponding $\frac{S(q,t)}{S(q,0)}$ spectra computed for the neat ring 20k PEO melt (Figure 6a2).
(b) In the L-10k blend, a totally different picture emerges. Compared to the pure 20k ring or the behavior of rings in the unentangled L-02k blend, the simulation results here indicate an initial rapid decay at short times but then a rather asymptotic and time-independent behavior which leads to plateau values for $\frac{S(q,t)}{S(q,0)}$ that depend on the momentum transfer *q*. The time scale of the fast initial decay depends on the wavenumber *q* but overall is seen to be between 30 and 100 ns, i.e., on the order of the entanglement time *τ*_e_ for entangled linear PEO melts at 413K, see Section 3.4 and Section 3.6 below. A closer inspection reveals that the initial fast decay is even steeper than the one recorded in the corresponding pure ring PEO-20k melt, implying more freedom for motion. According to Goossen et al. [21], at these short times, rings in the L-10k blend enjoy free 3-d Rouse motion in the tubes formed by the surrounding (moderately entangled, number of entanglements *Z* ≅ 5) L-10k linear chains. At later times, strong topological interactions (hindrance effects [21]) set in, which cause $\frac{S(q,t)}{S(q,0)}$ to cross over to time-independent plateau values exhibiting no sign of any further decay.
(c) In the longer L-20k blend, the dynamic structure factor exhibits the same qualitative behavior as in the L-10k blend. Again, we observe the rapid initial decay at very short times (on the order of 30 to 100 ns depending on the wavenumber *q*), followed by the time-independent behavior towards plateau values that are practically the same with those observed in the L-10k blend.
(d) The qualitative agreement between predicted and experimentally measured $\frac{S(q,t)}{S(q,0)}$ spectra in the L-02k and L-20k blends is excellent. The quantitative agreement, on the other hand, is fairly good but improves for larger *q* values (larger than approximately 0.10 Å^−1^). For lower *q* values the simulation results systematically overpredict the measured NSE data. We believe that any quantitative differences between simulated and experimentally measured spectra should be attributed to the united-atom nature of the force-field employed in the MD simulations.

Overall, our MD data provide solid support of the NSE findings of Goossen et al. [21] that the dynamics of ring molecules is enhanced in low molecular weight linear matrices but is dramatically slowed down in high molecular weight matrices where their diffusive motion is substantially hindered from the strong topological constraints imposed on their dynamics by the surrounding entangled linear chains.

### 3.4. Mean Square Displacement of Atomistic Segments

**Figure 7:**
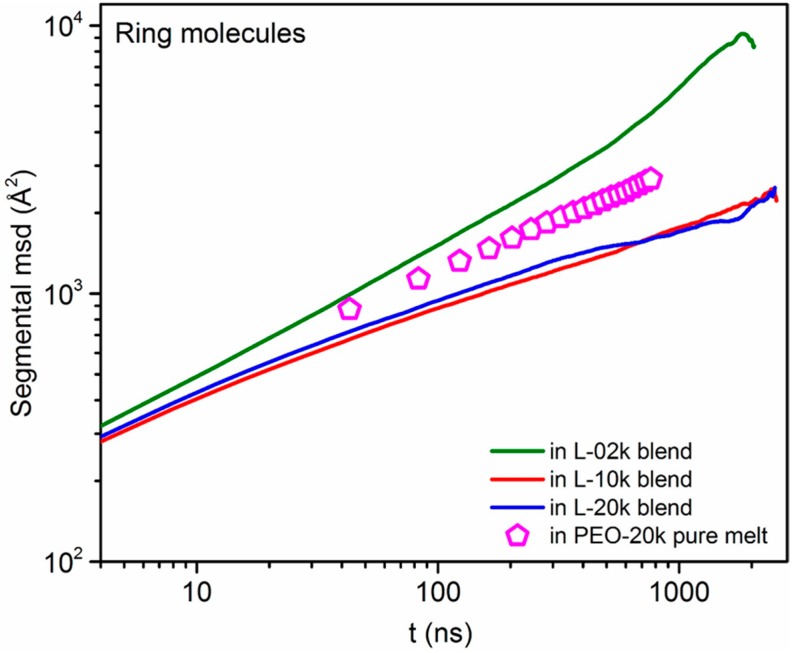
Log–log plot of the segmental mean-square displacement of ring PEO-20k molecules with time *t* in the three blends and in their own melt.

**Figure 8:**
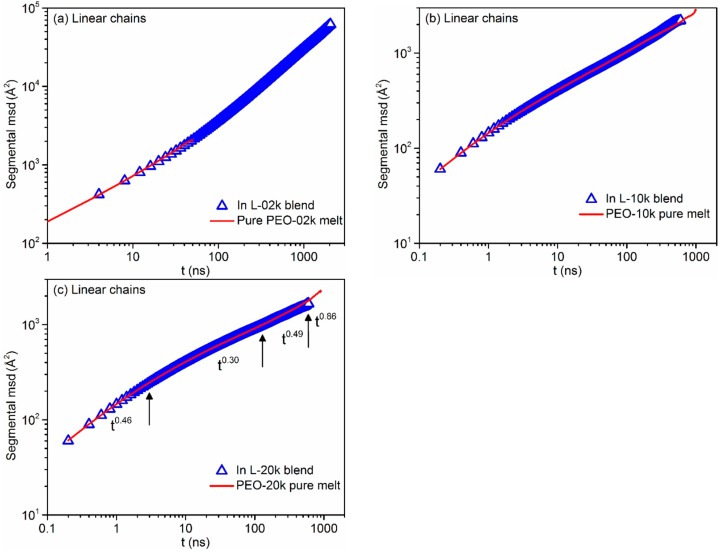
Log–log plot of the mean-square displacement of atomistic segments of linear chains with time *t* in: (**a**) the L-02k blend and the pure linear PEO-02k melt, (**b**) the L-10k blend and the pure linear PEO-10k melt, and (**c**) the L-20k blend and the pure linear PEO-20k.

Additional insight into the dynamics of the simulated ring–linear blends at a very small fraction of ring chains is obtained by analyzing the mean-square-displacement (msd) of atomistic segments separately for ring molecules and linear chains. The results (lines) are shown in a log–log plot in Figure 7 and Figure 8 together with the corresponding msd data for the pure melts (symbols). Clearly, in the L-02k blend, 20k-PEO ring molecules exhibit significantly faster segmental dynamics than in their own melt, which is consistent with our conclusions from the normal mode analysis that, in an unentangled linear chain matrix, rings perform unrestricted Rouse motion. In marked contrast, in the entangled matrices (L-10k and L-20k), the opposite effect is seen: here, the ring segmental motion is significantly slowed down, and is actually the same in the two blends. As we already discussed, our explanation for this is that it is due to strong threading from the long linear matrix chains [35,37]. We return to this issue in Section 3.6 below.

The situation is different for the segmental dynamics of linear chains in the three blends for which Figure 8 demonstrates that it is not affected at all by the presence of the few rings in the system. For all practical purposes, the segmental msd curves of linear chains in the three blends computed here from the MD simulations coincide with those obtained from the simulations with the corresponding neat linear melts of the same chain length. In Figure 8c, we can also distinguish the three characteristic breaks in the msd vs. *t* plot as predicted by the reptation theory [39]: the onset of tube constraints on segmental diffusion at short times, the Rouse-like diffusion combined with tube constraints at intermediate times, and the passage from the Rouse-like diffusion to reptation dynamics at longer time scales. Each of the three arrows in Figure 8c has been added by identifying the time at which the two straight lines (in the log–log plot) providing the best fits to the simulation data before and after the break cross each other. The different power exponents in the four different characteristic regimes are also indicated in the figure. Unfortunately, because of the use of multiple time origins in order to compute a smooth segmental msd-vs.-*t* curve and the fact that we only performed one MD simulation for each system, we could not come up with a strict calculation of error bars for these exponents. It is encouraging, however, that the values of these characteristic exponents are very similar to those already reported in the literature for other, moderately entangled, linear polymers such as polyethylene and *cis*-1,4-polybutadine [60,61,62,63].

### 3.5. Mean-Square-Displacement of Chains Centers-of-Mass

**Figure 9:**
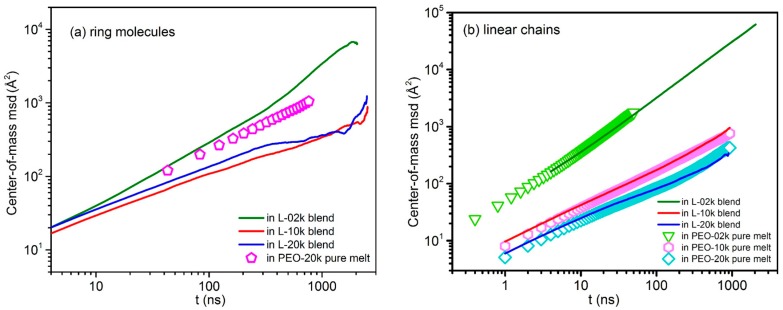
Log–log plot of the mean-square displacement of the centers-of-mass of ring molecules (**a**) and linear chains (**b**) with time *t* for all simulated systems.

Exactly the same conclusions are drawn by analyzing the simulation predictions for the msd of the centers-of-mass of the probe ring molecules and of the linear chains in the three blends. The results are shown in Figure 9 and support the findings discussed in the previous paragraphs that, in the L-02k blend, rings exhibit a faster mobility (faster ring center-of-mass diffusion) than in their pure ring melt. We also observe that, after approximately 2μs, ring motion in this blend has reached the regime of normal (Fickian) diffusion (msd ~*t*^1^), which allows us to compute the corresponding diffusion coefficient *D* of 20k PEO rings in this blend. The result is 0.50 ± 0.04 Å^2^/ns, in excellent agreement with the experimentally measured value of 0.49 Å^2^/ns by Goossen et al. [21]. Linear chains, on the other hand, in the three blends exhibit dynamics that is identical to that in their own neat melt.

Overall, our MD simulations support the idea that ring PEO dynamics in linear PEO melt matrices with molecular weight *M* of the same order of magnitude as the entanglement molecular weight *M*_e_ is accelerated compared to the pure melt, whereas in linear PEO matrices with molecular weight *M* well above *M*_e_ it is significantly slowed down.

### 3.6. Topological Analysis

The underlying topological structure [59,64] of the simulated blends can be analyzed by computing the number of threading events (penetrations) of a ring molecule by linear chains in the three blends (especially in the two entangled blends). This is a very important calculation, since previous studies [35] have shown that in mixtures of ring and linear chains, a linear chain can thread as many rings as its number of entanglements *Z* in its pure melt. First, we report that our topological analysis showed that all ring molecules in the three matrices (L-02k, L-10k and L-20k) are threaded by at least one linear chain. Second, we examined how many times (on average) a ring molecule is threaded by linear chains or other ring molecules. Actually, because of the very small concentration of rings in the blends, the average number of ring–ring threadings per ring molecule was found to be very small and this issue will not be discussed any further here.

**Figure 10:**
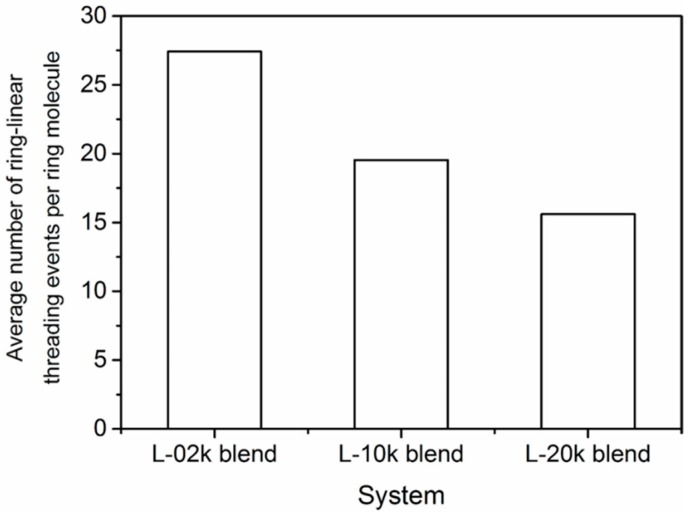
Average number of linear chains that thread a ring molecule in the three blends.

The results for the average number of ring–linear threadings per ring molecule are presented in Figure 10. Our topological analysis shows that PEO-20k ring molecules suffer significant threading in all blends: The average number of threadings per ring is approximately 27 in the L-02k bled, approximately 20 in the L-10k blend, and approximately 16 in the L-20k matrix. Clearly, the number of threading events per ring decreases as the chain length of linear chains increases, because, at constant weight fraction of the ring component, the corresponding relative number of linear chains decreases as well.

**Figure 11:**
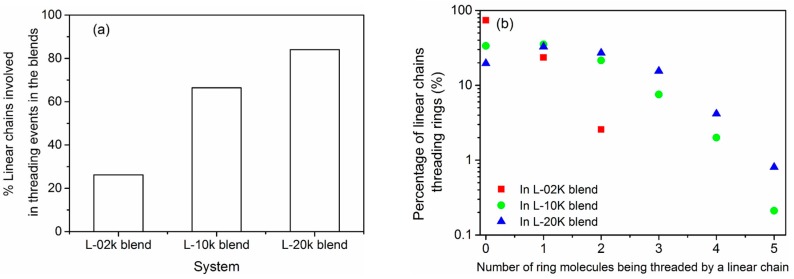
(**a**) Percentage of linear chains that are involved in threading events with ring molecules in the three blends; and (**b**) percentage of linear chains involved in multiple threading events.

We have also analyzed the percentage of linear molecules that participate in threading events with ring molecules, and the results are shown in Figure 11. We see that the percentage of linear chains that are computed to penetrate ring molecules increases substantially as their length increases, and the same with their fraction that is involved in multiple threadings. For example, in the L-10k and L-20k blends, a finite number of linear chains are seen to thread up to five rings, simultaneously. In the L-02k, on the other hand, the maximum number of rings that a linear chain can simultaneously thread is two. Furthermore, our geometric analysis showed that in all blends, there is no ring that remains unthreaded by linear molecules. Moreover, there is no ring that is simultaneously threaded by less than six linear chains.

**Figure 12:**
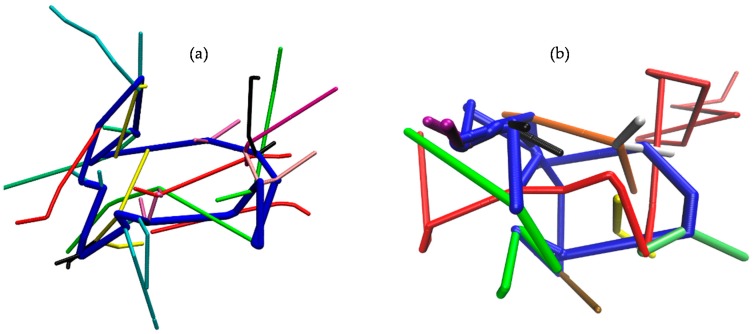
Examples of multiple threading. The snapshots have been taken from the combined geometric/topological analysis of: (**a**) the L-02k blend, and (**b**) the L-20k blend. (**a**) The blue ring molecule is simultaneously threaded by fifteen linear chains. (**b**) The blue ring chain is simultaneously threaded by ten linear chains (here, for simplicity, only the PP of the red linear chain is shown in full; for all other linear chains, only the part of their PP whose strands are involved in the threading is shown).

Typical snapshots from our geometric analysis demonstrating examples of multiple threadings are shown in Figure 12. In Figure 12a, our geometrical analysis identified a ring molecule (colored in blue) in the L-02k blend that is threaded simultaneously by fifteen linear chains. In Figure 12b, we show a snapshot from the geometric analysis with the L-20k melt where another ring molecule (also colored in blue) is simultaneously threaded by ten linear chains.

An important question raised by Goossen et al. [21] was what is the effect of the guest ring molecules on the tube diameter *d*_t_ of the host linear chains in the three blends. The tube diameter is a very difficult quantity to calculate and, in the literature, is typically taken to be equal to the corresponding Kuhn step length of the PP. However, Stephanou et al. [63] have proposed a method that leads directly to the calculation of the tube diameter *d_t_* by monitoring the mean-square displacement (msd) of the innermost chain segments $\phi (t)=\langle {\left(r_{n}(t)-r_{n}(0)\right)}^{2}\rangle$ vs. time *t* and observing the 1st break signaling the onset of tube constraints on segmental dynamics. The tube diameter *d*_t_ is then estimated as $d_{t}=2\sqrt{\phi \left(t^{*}\right)}$ where $\phi (t^{*})$ is the value of ϕ(t) at the time $t=t^{*}$ where the slope of ϕ(t) starts to change as the msd leaves the initial *t*^1/2^ regime to enter the next *t*^1/4^ regime. The method was applied by Stephanou et al. [63] to melts of entangled linear polyethylene and *cis*-1,4-polybutadiene melts with remarkable success. It was also found that the usual approach to take the tube radius equal to the Kuhn step length of the PP overestimates *d*_t_ by 15%–30%.

**Figure 13:**
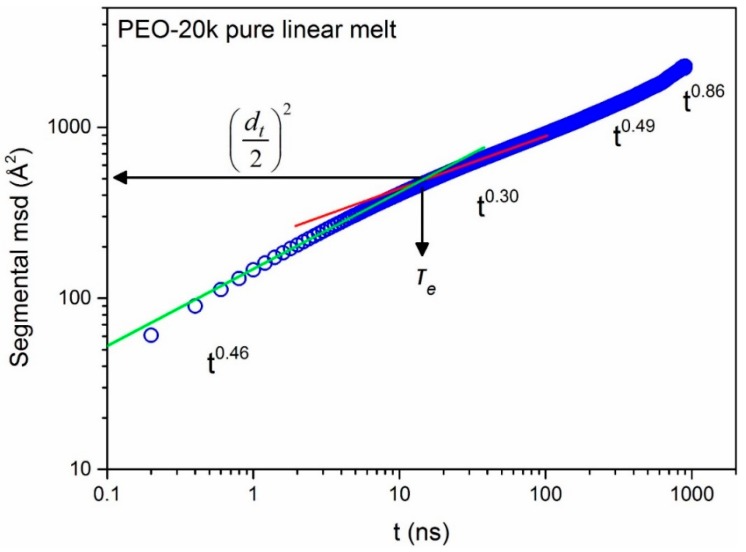
Calculation of the tube diameter *d_t_* based on the segmental mean-square displacement of the innermost chain segments $\phi (t)=\langle {\left(r_{n}(t)-r_{n}(0)\right)}^{2}\rangle$ versus time *t*. The tube diameter is estimated as $d_{t}=2\sqrt{\phi \left(t^{*}\right)}$ where $\phi (t^{*})$ is computed at time $t=t^{*}$ where the first break is observed as segments leave the initial *t*^1/2^ regime to enter the next *t*^1/4^ regime.

A typical calculation of *d*_t_ with the Stephanou et al. [63] method is shown in Figure 13; it refers to the pure 20k linear PEO melt. We see that as the linear chain segments “feel” the tube constraints, a break in their displacement appears; the resulting value of the tube diameter then is *d*_t_ = 40 ± 5 Å. Given that the segmental msd’s of 20k linear chains are identical in the L-20k blend and in their own melt (see Figure 7), we understand that the same value of *d*_t_ should characterize the L-20k blend as well. For the 10k linear chains, the corresponding prediction for the tube diameter (both in the L-10k blend and in their melt) is *d*_t_ = 33 ± 3 Å. Our estimate (*d*_t_ = 40 ± 5 Å) for the tube diameter in the L-20k blend is in excellent agreement with the value reported by Goossen et al. [21] (*d*_t_ = 42 ± 1 Å) through a description of tube constraints on their measured $\frac{S(q,t)}{S(q,0)}$ at times beyond *τ*_e_ with a Gaussian distribution. Equally excellent is the agreement between MD data and NSE measurements as far as the order of magnitude of the entanglement time *τ*_e_ (~20 ns) is concerned.

## 4. Conclusions

In summary, our detailed MD simulations provide convincing evidence that the dynamics of a small number of long ring PEO molecules immersed in a host matrix of linear PEO chains is controlled by the length of the host linear chains. In matrices of unentangled linear chains, the probe rings diffuse faster and assume more expanded conformations than in their own melt. In striking contrast, in matrices of long, entangled linear chains, their dynamics is dramatically slowed down. For example, the spectra of their dynamic structure factor exhibit a steep decay at short times (up to the entanglement time *τ*_e_ of linear PEO at the same temperature) but then become practically time-independent approaching constant asymptotic values that are different for different wave-vectors and depend strongly on the molecular weight of the host linear chains.

A subsequent geometric analysis of topological constraints in the blends revealed significant threading of all rings molecules by the host linear chains. In almost all cases, each ring was found to be simultaneously threaded by a large number of linear chains. This implies that its dynamics at times longer than *τ*_e_ should be completely dictated by ring–linear disentanglement events, which explains the asymptotic values exhibited by the dynamics structure factor spectra at times longer than approximately a few *τ*_e_. In striking contrast, our topological analysis did not indicate any appreciable effect of the few rings on the average size or the statistics of the network of primitive paths of the host linear chains. A direct calculation of the tube diameter gave a value which was seen to coincide with the value extracted indirectly from the measured $\frac{S(q,t)}{S(q,0)}$ spectra [21] by describing tube constraints with a Gaussian distribution of obstacles. MD simulation predictions and NSE measurements agree also on the value of the entanglement time *τ*_e_ for linear PEO (~20 ns at 413 K).

In the future, we plan to investigate the effect of the concentration (weight fraction) of ring molecules on the dynamics of the host linear chains. We also plan to extend the simulations to matrices of longer linear chains whose relaxation time will be significantly longer than that of the ring molecules, implying the presence of a frozen network of obstacles. We further mention that our analysis has focused primarily on ring PEO molecules, however we expect it to have important implications for a variety of problems in Soft Matter Physics where semiflexible ring polymeric structures are involved, such as the organization of chromatin in the cell nucleus, the separation of DNA, and the stabilization of protein structures [65,66,67,68,69].

## Figures and Tables

**Table 1:** Simulated poly(ethylene oxide) (PEO) blend systems and number of ring and linear PEO molecules in each one of them.

System	Host matrix	Number of ring PEO-20k molecules	Number of linear PEO chains	Volume fraction of ring molecules
1	L-02k	8	720	0.1
2	L-10k	8	144	0.1
3	L-20k	8	72	0.1

**Table 2:** MD predictions for $\langle R_{g,R}^{2}\rangle$, $\langle R_{g,L}^{2}\rangle$ , $\langle R_{d}^{2}\rangle$ and $\langle R_{\mathrm{ee}}^{2}\rangle$ in all simulated PEO systems (pure and blends).

System	Ring molecules		Linear chains		
	$\langle R_{g,R}^{2}\rangle$ (Å^2^)	$\langle R_{d}^{2}\rangle$ (Å^2^)	$\langle R_{g,L}^{2}\rangle$ (Å^2^)	$\langle R_{\mathrm{ee}}^{2}\rangle$ (Å^2^)	$\langle R_{\mathrm{ee}}^{2}\rangle /\langle R_{g,L}^{2}\rangle$
02k pure linear melt	-	-	235 ± 55	1475 ± 140	6.3 ± 0.7
10k pure linear melt	-	-	1410 ± 180	8420 ± 310	6.0 ± 0.8
20k pure linear melt	-	-	2660 ± 320	17,015 ± 930	6.4 ± 1.0
L-02k blend	1150 ± 120	3040 ± 260	240 ± 25	1525 ± 105	6.3 ± 1.4
L-10k blend	885 ± 105	2320 ± 240	1380 ± 85	8160 ± 220	5.9 ± 08
L-20k blend	815 ± 110	2055 ± 260	2975 ± 185	16,925 ± 500	5.7 ± 0.8
20k pure ring melt	825 ± 63	2250 ± 120	-	-	-

**Table 3:** MD predictions for the packing length $\tilde{l}$ and the persistence length $l_{p}$ of all simulated PEO systems (pure and blends).

System	$\tilde{l}$ of ring molecules (Å)	$l_{p}$ of ring molecules (Å)	$\tilde{l}$ of linear chains (Å)	$l_{p}$ of linear chains (Å)
02k pure linear melt			12 ± 3	6.7 ± 0.4
10k pure linear melt			12 ± 3	6.7 ± 0.4
20k pure linear melt			11 ± 3	6.7 ± 0.4
20k pure ring melt	35 ± 3	6.1 ± 0.4		
L-02k blend	28 ± 3	6.1 ± 0.4	13 ± 3	6.7 ± 0.4
L-10k blend	31 ± 3	6.1 ± 0.4	12 ± 3	6.7 ± 0.4
L-20k blend	38 ± 4	6.1 ± 0.4	12 ± 3	6.7 ± 0.4
